# Combinatorial Library Based Engineering of *Candida antarctica* Lipase A for Enantioselective Transacylation of *sec*-Alcohols in Organic Solvent**

**DOI:** 10.1002/anie.201410675

**Published:** 2015-02-09

**Authors:** Ylva Wikmark, Maria Svedendahl Humble, Jan-E Bäckvall

**Affiliations:** Department of Organic Chemistry, Arrhenius Laboratory, Stockholm University10691 Stockholm (Sweden); Industrial Biotechnology, School of Biotechnology, Albanova University CenterRoyal Institute of Technology (KTH), 10691 Stockholm (Sweden)

**Keywords:** biocatalysis, kinetic resolution, lipase A, protein engineering, secondary alcohols

## Abstract

A method for determining lipase enantioselectivity in the transacylation of *sec*-alcohols in organic solvent was developed. The method was applied to a model library of *Candida antarctica* lipase A (CalA) variants for improved enantioselectivity (*E* values) in the kinetic resolution of 1-phenylethanol in isooctane. A focused combinatorial gene library simultaneously targeting seven positions in the enzyme active site was designed. Enzyme variants were immobilized on nickel-coated 96-well microtiter plates through a histidine tag (His_6_-tag), screened for transacylation of 1-phenylethanol in isooctane, and analyzed by GC. The highest enantioselectivity was shown by the double mutant Y93L/L367I. This enzyme variant gave an *E* value of 100 (*R*), which is a dramatic improvement on the wild-type CalA (*E*=3). This variant also showed high to excellent enantioselectivity for other secondary alcohols tested.

The general demand for stereoselective synthetic methods and environmentally compatible industrial chemical processes is increasing. This has led to an increased demand for enzymes that can be used as catalysts for various purposes.[1,2] To date, lipases are among the most used enzymes for industrial biocatalytic applications.[3,4] Lipases generally show high substrate and reaction promiscuity in combination with high stereoselectivity.

Our research group has been involved in the development of dynamic kinetic resolution (DKR),[5–7] in which lipases are combined with transition metals. Efficient systems have been obtained that allow the transformation of racemic alcohols (and amines) into enantiomerically pure products in close to quantitative yields based on the racemate. In these reactions, the lipase catalyzes a transacylation in an organic solvent and the metal complex racemizes the slow-reacting enantiomer of the alcohol (or amine).

To further improve and extend the scope of DKR reactions, more efficient and more enantioselective enzymes with broad substrate scope are required. One problem is that such enzymes may not be available in the natural collection of lipases. A solution to this problem is to modify natural lipases through engineering/directed evolution.

Evolution of enzymes through the generation of large libraries with subsequent screening and selection is the most efficient method for obtaining new enzyme variants with improved properties.[8–10] There are many examples in which the stereoselectivity of lipases has been significantly improved by using this approach.[8,11–18] Although a method for microtiter-plate screening in organic solvent has been reported, this method was not tested for enantioselectivity.[19] To date, all screening studies on lipase libraries for increased enantioselectivity have dealt with the hydrolysis of esters in an aqueous medium. Consequently, library-based engineering has never been used for improving the DKR scope for *sec*-alcohols, since these substrates involve transacylation in organic solvent. In order to produce the desired enzyme variant, it is necessary to mimic the true reaction conditions according to Arnold’s statement: “you get what you screen for”.[8a] There are several reported examples of solvent dependence for enzyme selectivity,[20,21] and even reversed enantioselectivity between water and organic solvent has been described.[21]

Herein, we report a method that enables the screening of a lipase library for enantioselective transacylation in an organic solvent. Normally, supernatants containing the protein of interest are directly used in enzyme-library screening assays. In this work, the use of a standard His_6_-tag immobilization technique provided one-step simultaneous immobilization and purification of the enzyme library on microtiter plates. This method allows the solvent to be changed from water to isooctane, as well as the screening of purified enzyme preparations.

Lipases A and B from *Candida antarctica* are two biocatalysts that have found widespread applications in organic transformations, and both enzymes work very well in a range of organic solvents.[22] The enzyme *Candida antarctica* lipase A (CalA) has been employed to a lesser extent compared to *Candida antarctica* lipase B (CalB). CalA catalyzes the transacylation of *sec*-alcohols in organic solvents at activities comparable to those of CalB,[23] but shows low or no enantioselectivity for small *sec*-alcohols such as 1-phenylethanol (*E*=3).[24] CalA shows a naturally high thermostability, even in its free form,[23–25] and it has a rather large active site that allows the transacylation of tertiary alcohols.[26] We have previously demonstrated that CalA can be modified through engineering/directed evolution for the enantioselective hydrolysis of esters in water.[17,18,27] In the present work, we used CalA as a test enzyme for improvement of the enantioselectivity of transacylation in an organic solvent.

As in all lipases, the active site of CalA is made up of two main substrate binding pockets; an acyl binding pocket and a nucleophile (or alcohol) binding site. The enzyme exposes its active site through interfacial activation.[28] Crystal structures of CalA[29,30] reveal a narrow acyl binding tunnel, a wider nucleophile binding cleft, and a flexible lid.[31] The wide nucleophile binding pocket may be the reason why CalA shows low enantioselectivity with small secondary alcohols. Hypothetically, decreasing the size of the nucleophile binding pocket should increase the enantioselectivity of the enzyme with smaller secondary alcohols.

Large gene libraries created by random mutagenesis often show low hit ratios. For improved enantioselectivity, mutations around the active site have proven to be the most influential and in these cases, focused combinatorial gene libraries with simultaneous variation of a few amino acid residues have proven to be successful.[9,11,17,18] We recently reported an efficient improvement in the enantioselectivity of CalA for the hydrolysis of bulky esters in buffer, where nine amino acid positions around the active site were varied simultaneously. This combinatorial method accounts for synergistic effects. Using 2–4 amino acids at each site yielded a library of 1024 enzyme variants.[17,32]

In the present study, this combinatorial approach[17] was used to create a focused enzyme library. The library obtained was immobilized on microtiter plates through chelation of a His_6_-tag and then screened for enantioselective transacylation in an organic solvent. Seven amino acid residues in the active site were targeted and varied simultaneously. The combinatorial library design was guided by molecular modeling (Figure 1). The closed form of the crystal structure of CalA (PDB ID: 2VEO) was previously opened by a 10 ns molecular dynamics simulation.[18] By using the YASARA software[33] starting from the catalytic Ser184, tetrahedral intermediates of (*R*)- and (*S*)-1-phenylethyl butyrate were built into the open structure. Amino acid residues within 5 Å of the tetrahedral intermediates were identified. Seven positions were selected to be included in the focused combinatorial library (Table 1). These positions were to be varied to one or two other amino acids to yield an enzyme library of 648 variants. The residues were chosen to modify the space available in the active site.

**Figure 1:**
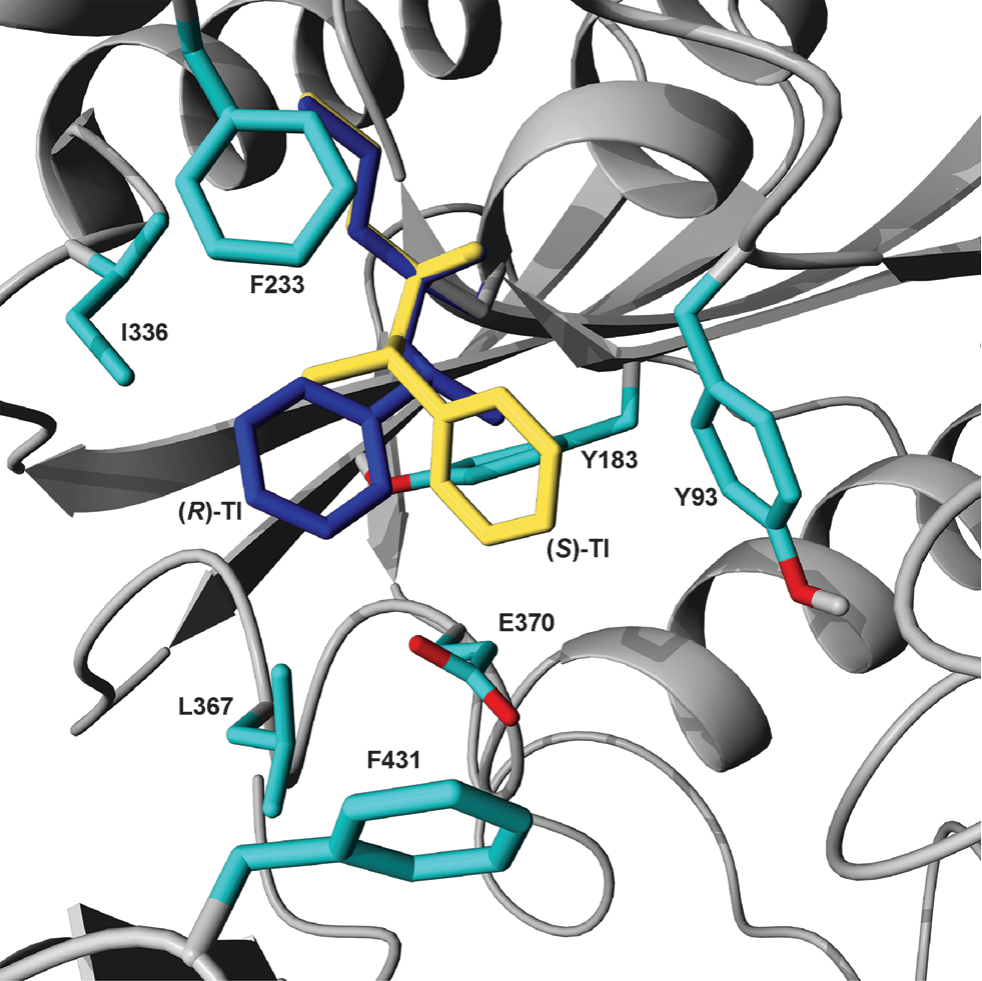
Close-up view of the active site of CalA. Amino acid residues included in the CalA library (Table 1) are shown as turquoise stick models, while the overall structure is shown as a gray ribbon diagram. Tetrahedral intermediates (TI) of the product, 1-phenylethyl butyrate, are shown in dark blue for the (*R*)-enantiomer and in yellow for the (*S*)-enantiomer. The molecular graphics were created by using the YASARA software.[33]

**Table 1:** The amino acid (Aa) residues included in the CalA library.

Aa_position_	Aa_wild−type_	Aa_library_
93	Y	A or L or Y
183	Y	Y or W
233	F	A or F
336	I	F or I or L
367	L	F or I or L
370	E	E or F or L
431	F	F or L

The gene library was created through overlap-extension PCR with seven small gene fragments. The gene fragments were fused together through PCR to give one full-length gene sequence. This polynucleotide was used as a megaprimer in a new PCR reaction with a gene–plasmid construct containing a His_6_ affinity tag in the N-terminus as the mother template. The product was transformed into *Pichia pastoris* X33, which was then grown on agar plates.

To exclude variants lacking lipase activity, the agar plates contained tributyrin [0.5 % (v/v)]. The use of tributyrin enables visible identification of colonies containing active lipase through the formation of halos where the tributyrin is hydrolyzed.[34] About 25–30 % of all colonies showed lipase activity after four days of incubation at 29 °C. This pre-screening step reduced the library size substantially and the accuracy of the selection was verified with a small fraction of the library.

In the library screening, individual colonies were expressed in 96-well deep-well plates (8 days, 29 °C, 250 rpm). The plasmid used, pBGP1, enables secretion of the protein into the supernatant.[27,35]

Supernatants were transferred to Nunc immobilizer Ni-Chelate F96 microtiter plates from ThermoScientific. The nickel coating of the microtiter plates enables the coordination of His_6_-tagged proteins to the walls of each well. After enzyme immobilization on the plate, the supernatant was removed. To remove residual water from the plate, each plate was washed three times with isooctane and air dried.

A screening solution of isooctane containing vinyl butyrate (200 mm), 1-phenylethanol (20 mm), and dodecane (20 mm, as internal standard) was added to the plates containing the immobilized lipase variants (Scheme 1). After 2.5 h, samples were transferred to a second 96-well microtiter plate. The plate was then screened by chiral gas chromatography (GC-FID). The total time to screen through one 96-well plate was around 24 h when using a method that takes 14 min per analysis. Variants showing enantioselectivity (*ee*_p_) over 95 % were determined to be hits. In total, 17 hits were cultivated in 50 mL scale for further characterization (Table S1 in the Supporting Information).

**Scheme 1:**
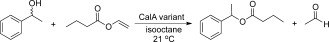
The model transacylation reaction applied to explore the enantiospecificity of CalA variants in the kinetic resolution of 1-phenylethanol in organic solvent at 21 °C.

The 17 enzyme variants considered to be possible hits were sequenced, but only three different variants were identified (Y93A/F233A/L367I, Y93L/L367I, and Y93L/L367I/G387E). However, the mutation G387E (G387 is positioned on the enzyme surface) was spontaneous since it was not included in the library.

Enzyme kinetics were measured for wild-type CalA and the Y93L/L367I variant. Pseudo-one-substrate conditions were applied for the model reaction by varying the concentration of 1-phenylethanol in a microtiter plate. The method involving His_6_-tag binding gives approximately equal amounts of enzyme in each well, which gives a reliable comparison of the activity of the variants. The Y93L/L367I variant showed increased apparent *K*_M_ and *K*_i_ values compared to the wild-type enzyme. Wild-type CalA gives a low apparent *K*_i_ value of 30 mm and the corresponding value shown by the Y93L/L367I variant is improved 2.5-fold (81 mm; Table 2). An improved inhibition constant is beneficial for further applications in DKR,[36,37] in which higher substrate concentrations are preferred. Variant Y93A/F233A/L367I showed varying results, thus indicating a mix of different enzyme variants. This variant was not further characterized.

**Table 2:** Apparent kinetic parameters for wild-type CalA and the Y93L/L367I variant determined for the kinetic resolution of 1-phenylethanol (model reaction) under pseudo-one-substrate conditions at 21 °C.^[a]^

CalA variant	*K*_M,app_^[b]^ [mm]	*K*_i,app_^[b]^ [mm]	*E*^[c]^
Wild-type	22	30	3
Y93L/L367I	45	81	96

[a] The concentration of vinyl butyrate was kept constant at 500 mm while varying the concentration of 1-phenylethanol (3-200 mm). Undecane or dodecane (20 mm) was used as an internal standard. [b] The kinetic constants were derived from non-linear regression using the Michaelis–Menten equation with substrate inhibition. [c] Mean values of 3–6 reactions with 20 mm 1-phenylethanol for the wild-type and 50 mm 1-phenylethanol for Y93L/L367I.

The enantiospecificity and substrate scope of variant Y93L/L367I for a number of *sec*-alcohols were determined and compared to those of wild-type CalA. Enzyme variant Y93L/L367I showed improved enantioselectivity for six out of the seven alcohols evaluated (Table 3). For 1-phenylethanol (entry 1), *p*-substituted 1-phenylethanol (entries 3 and 4), and 1-phenylchlorohydrin (entry 7), a significant increase in the *E* value was obtained.

**Table 3:** Enantioselectivity of wild-type CalA and the Y93L/L367I variant determined for the kinetic resolution of various secondary alcohols by transacylation in isooctane at 21 °C.[a]

Entry	Alcohol	*E*_wild-type_	*E*_Y93L/L367I_
1	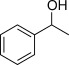	3	100
2	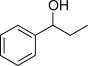	5	20
3	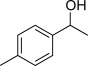	30	>300
4	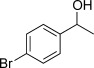	70	>300
5	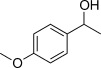	70	100
6	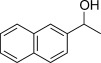	32	23
7	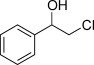	4	100

[a] Reaction conditions: 200 mm vinyl butyrate, 20 mm alcohol, and 20 mm dodecane in isooctane at 21 °C. The enzyme was immobilized on Nunc 96-well nickel-coated microtiter plates.

The effect of each mutation in the Y93L/L367I variant on the enantioselectivity of kinetic resolution in the model reaction was explored by applying variants with single point mutations (Y93L and L367I). These variants were created by site-directed mutagenesis, cultivated, immobilized on 96-well nickel-coated microtiter plates, and screened with the model reaction (Scheme 1). The screening results show that the two mutations are synergistic and that the Y93L mutation has the larger influence on the enantiospecificity.

The effects of the mutations on enantioselectivity were further explored through molecular modeling by using a tetrahedral intermediate model (Figure S1 in the Supporting Information). The structure was prepared as described above (by using the YASARA software). Double mutant Y93L/L367I and the two single mutants (Y93L and L367I) were created by swapping the specific amino acid residues, followed by energy minimizations. The (*R*)- or (*S*)-configurations of the tetrahedral intermediate in the different variant structures were compared through alignment to the corresponding configurations in the wild-type enzyme by using MUSTANG.[38]

The alignments showed that the mutations induce movement of the phenyl groups as a result of steric hindrance. These movements were measured and the resulting structures compared to the wild-type structure (Table S3). Mutation Y93L mainly affects the phenyl group of the tetrahedral intermediate in the (*S*)-configuration, while mutation L367I mainly affects the corresponding phenyl group in the (*R*)-configuration. According to the molecular modeling results, mutation Y93L/L367I affects the tetrahedral intermediate in the (*R*)-configuration in a positive manner to a higher extent than the intermediate in the (*S*)-configuration, which has to move further away (see the Supporting Information). However, the important hydrogen-bond network between the tetrahedral substrate intermediate [(*R*) or (*S*)] and the oxyanion hole and His366 was not affected by the mutations. These molecular modeling results (compiled in the Supporting Information) confirm and follow the trend of the experimentally determined results for the three enzyme variants (Table 3, entry 1 and Table 4).

**Table 4:** Enantioselectivity of CalA single mutants determined for the kinetic resolution of 1-phenylethanol (model reaction).[a]

CalA variant	*ee*_s_ [%]	*ee*_p_ [%]	Conversion [%]	*E*
Y93L	62	86	44	25
L367I	12	86	25	4

[a] Reaction conditions: 200 mm vinyl butyrate, 20 mm 1-phenylethanol, 20 mm dodecane in isooctane at 21 °C. The enzyme was immobilized on 96-well nickel-coated microtiter plates.

In conclusion, the reported method is a powerful approach for discovering new enantioselective biocatalysts for the transacylation of alcohols in organic solvents. By using the standard His_6_-tag binding technique, simultaneous immobilization and purification of the whole enzyme library was achieved. The method is general and should be applicable to other enzymes for improving enantioselectivity and activity. Furthermore, the screening method is easy to handle, can be performed within a reasonable time, and is not labor intensive. The whole screening procedure is done in 28.5 h/plate: 2 h (immobilization)+2.5 h (biocatalysis)+24 h (GC measurement).

By using this screening method, a lipase variant showing improved enantioselectivity in the transacylation of 1-phenylethanol was found. The enantioselectivity increased from 3 (*R*) to 100 (*R*). In addition, this enzyme variant showed increased *K*_M_ and *K*_i_ values compared to the wild-type enzyme. The improvement in the *K*_i_ value, from 30 to 85 mm, makes the enzyme more useful for synthetic applications. The enzyme variant showed significantly improved enantioselectivity for several secondary alcohols. The dramatically increased enantioselectivity was confirmed by molecular modeling. The highly combinatorial approach for increasing enantioselectivity reported by our group[17] was thus again proven to be successful.
