# Supplementary material for: Combinatorial Library Based Engineering of Candida antarctica Lipase A for Enantioselective Transacylation of sec-Alcohols in Organic Solvent
Source: Angew Chem Int Ed Engl. 2015 Feb 9;54(14):4284–8. doi: 10.1002/anie.201410675 (PMC4471580; doi:10.1002/anie.201410675)
Supplement: Supplementary file 1 [file anie0054-4284-sd1.pdf]

Angewandte Chemie
Eine Zeitschrift der Gesellschaft Deutscher Chemiker

Supporting Information

Combinatorial Library Based Engineering of *Candida antarctica* Lipase A for Enantioselective Transacylation of *sec*-Alcohols in Organic Solvent**

Ylva Wikmark, Maria Svedendahl Humble, and Jan-E. Bäckvall**

anie_201410675_sm_miscellaneous_information.pdf

Chemicals, enzymes and molecular biology kits: 2
Equipment: 2
Media recepies: 2
Selection of mutation points by molecular modeling: 2
pBGP1-CalA plasmid: 2
Primers: 2
Creation of library gene fragment: 3
Preparation of enzyme library: 4
Organic solvent screening: 4
GC screen: 5
Sequencing hits: 5
Exploring hit mutations: 5
Sequencing hits: 6
Determination of kinetic parameters, K_M and K_I: 7
Exploring hit mutations by molecular modeling: 7
References: 8

Chemicals, enzymes and molecular biology kits:

All chemicals were purchased from Sigma-Aldrich, except Zeocin™ that was purchased from Invitrogen. Phusion Hot Start II DNA polymerase and FastDigest DpNI was purchased from Thermo Fischer Scientific Inc. Wizard® SV Gel and PCR product clean up PureYield system and Bacterial plasmid preparation kit was purchased from Promega. An E.Z.N.A.® Yeast Prep Kit was supplied from Omega-Bio Tek.

Equipment:

Bio-Rad Micropulser was applied for electroporation using electroporation cuvettes (0.2 mm) from Bio-Rad. GC analyses were performed on a Varian GC 3900 using an IVADEX-1 chiral column from IVA Analysentechnik.

Media recipes:

For bacterial cultivation; LB medium (10 g/L tryptone, 5 g/L yeast extract, 5 g/L NaCl, pH 7.0) and 50 µg/ml carbenicillin was utilized. Cultivation and protein expression in *Pichia pastoris* was performed in YP medium (20 g/L peptone, 10 g/L yeast extract), 10 % 200 g/L dextrose, 100 µg/ml carbenicillin and 100 µg/ml Zeocin.

Selection of mutation points by molecular modeling:

A library of CalA was designed based on knowledge from molecular modeling. The crystal structure of CalA[1] (2veo.pdb) in closed form[2] was previously opened by a 10 ns molecular dynamics simulation.[3] Starting from the catalytic Ser184, tetrahedral intermediates of the model reaction 1-phenylethyl butyrate (Figure S1) in both (*S*)- or (*R*)-configuration was built into the open structure using the Yasara software (www.yasara.org) [4] and further evaluated separately. The structures were energy minimized using Amber99 force field,V[5] 7,86 Å force cut-off point and particle mesh Ewald algorithm[6] to treat long range electrostatic interactions. After removal of conformational stress by a short steepest descent minimization, the procedure continued by simulated annealing (timestep: 2 fs, atom velocities were scaled down by 0.9 every tenth step) until convergence was reached, that is, the energy improved by less than 0.1 % during 200 steps. The structures were step-wise energy minimized, by firstly keeping all but the tetrahedral intermediate fixed followed by allowing everything to be free. Amino acid residues closer than 5 Å to the tetrahedral intermediates were identified. Seven amino acid residues (93, 183, 233, 336, 367, 370, 431) were selected to be included in a focused combinatorial library.

pBGP1-CalA plasmid:

The construction of the pBGP1-CalA plasmid has been described previously.[2] The template vector pBGP1 is an episomally replicating plasmid, useful for constitutively expressed enzyme libraries. [7] The α-factor secretion signal in the vector allows secretion of the produced enzyme into the supernatant.

Primers:

The following primers were used to construct the gene library. Primers are written in 5´-3´ and were ordered from Eurofins MWG Operon.

1a_f	CGT ACC AGG TCT ACG AGG ATG CCA CGG
1a_r	CCG TGG CAT CCT CGT AGA CCT GGT ACG
1b_f	CGT ACC AGG TCT TGG AGG ATG CCA CGG

1b_r	GCC GTG GCA TCC TCC AAG ACC TGG TAC
1c_f	CGT ACC AGG TCG CTG AGG ATG CCA CG
1c_r	CGT GGC ATC CTC AGC GAC CTG GTA CG
2a_f	CGC TCT TGA GGG CTA CAG TGG CGG
2a_r	CCG CCA CTG TAG CCC TCA AGA GCG
2b_f	CGC TCT TGA GGG CTG GAG TGG CGG
2b_r	CCG CCA CTC CAG CCC TCA AGA GCG
3a_f	CCC TTC GCC GGC TTT GCC CTG GC
3a_r	GCC AGG GCA AAG CCG GCG AAG GG
3b_f	CCT TCG CCG GCG CTG CCC TGG
3b_r	CCA GGG CAG CGC CGG CGA AGG
4_f	CGA TCC CCG ACG AGW TWG TGC CGT ACC
4_r	GGT ACG GCA CWA WCT CGT CGG GGA TCG
5a_f	GCC GAG CAC WTW ACC GCC TTK ATC TTT GG
5a_r	CCA AAG ATM AAG GCG GTW AWG TGC TCG GC
5b_f	GCC GAG CAC WTW ACC GCC GAA ATC TTT GG
5b_r	CCA AAG ATT TCG GCG GTW AWG TGC TCG GC
6_f	GCA GAG TGC GTT KGG CAA GCC CTT TGG C
6_r	GCC AAA GGG CTT GCC MAA CGC ACT CTG C
Anchor_f	CTGTAGCGGCCGCGCAAGAGGATCCATCTCTCGAGAAGAGAGAGG
Anchor_r	GTCGAGCGATATCGGTACCAAGCTCGTTCTAGACTAAGGTGGTGT

Degenerative nucleotide designation: K=T,G; M=A,C; W=A,T

Creation of library gene fragment:[8]

For cases where degenerate nucleotides did not fit the amino acid codons required, the following primers containing the same mutation point was premixed in appropriate ratios:

1_f: (1a_f, 1b_f and 1c_f) , 1_r: (1a_r, 1b_r and 1c_r), 2_f: (2a_f and 2b_f), 2_r: (2a_r, 2b_r), 3_f:(3a_f and 3b_f), 3_r: (3a_r and 3b_r), 5_f: (5a_f and 5b_f), 5_r: (5a_r and 5b_r)

The following primer pairs were applied to create overlapping gene fragments:

Fragment 1: Anchor_f and 1_r, Fragment 2: 1_f and 2_r, Fragment 3: 2_f and 3_r, Fragment 4: 3_f and 4_r, Fragment 5: 4_f and 5_r, Fragment 6: 5_f and 6_r, Fragment 7: 6_f and Anchor_r

PCR reactions:

Production of gene fragments: Each fragment was amplified in separate tubes. Each tube contained; 1X GC buffer, 10 ng pBGP1-CalA, 50 pmol of each primer pair, 200 µM of each dNTP, 1 U Phusion Hot Start II DNA polymerase, adjusted to 100 µL (MQ-H_2O). The reaction was started by denaturation at 96 °C for 2 min; then 24 cycles of 98 °C for 10 s, 72 °C (0.5 °C/cycle) for 30 s, 72 °C for 45 s; then 10 cycles of 98 °C for 10 s, 58 °C for 30 s, 72 °C for 2 min and a final elongation at 72 °C for 10 min. The PCR fragments were cleaned up by the Promega Wizard® SV gel and PCR clean up system. The concentration was determined by agarose gel densitometry.

Production of a full length gene fragment: Overlap Extension-PCR: In a tube adjusted to 100 µL (MQ-H_2O), 1X GC buffer, 30 fmol of every fragment, 200 µMol of each dNTP, and 1 U Phusion Hot Start II DNA polymerase was mixed. The reaction was started at 98 °C for 30 s, the cycled as following; 16 cycles of 98 °C for 10 s, 68 °C (−0.5°/cycle) for 30 s, 72 °C for 1 min and then hold at 4 °C for the

addition of amplification primers (50 pmol each), hereafter the program continued with 98 °C for 30 s, then subjected to 16 cycles of 98 °C for 30 s, 68 °C (−0.5°⁄cycle) for 30 s, 72 °C for 1 min, and then a final elongation of 72 °C for 10 min. The PCR fragments were cleaned up by using the Promega Wizard® SV gel and PCR clean up system. The concentration was determined by agarose gel densitometry and used as a megaprimer in the amplification of pBGP1-CalAH$_{6.}$

Amplification of gene-vector construct with the multimutated genefragment as megaprimer: In a tube adjusted to 100 μL (MQ-H_2O), 1X GC buffer, 100 fmol of the megaprimer, 200 μmol of each dNTP, 1 μL miniprep (pBGP1-CalAH$_6$) and 1 U Phusion Hot Start II DNA polymerase was mixed 95 °C for 2 min, then subjected to 30 cycles of 98 °C for 10 s, 63 °C (± 5 °) for 30 s, 72 °C for 3 min followed by final elongation at 72 °C for 10 min. The PCR fragments were cleaned up by the Promega Wizard® SV gel and PCR clean up system and digested with DpnI Fast Digest (3x additions)

Preparation of enzyme library

Transformation into competent DH5α cells: Competent cells were made heat-shock competent and stored in -80 °C until use.[9] Aliquots (100 µl) of the competent cells were thawed on ice. PCR product (1-5 µl) was added and gently mixed with the cells. The mixture was then left on ice for 30 min. The cells were heat shocked (42 °C, 45 s) in order for uptake of the plasmid to occur, held on ice for two minutes, where after SOC media (300 µl) was added. The cells were incubated in an orbital shaker (45-50 min, 37 °C, 250 rpm) after which 100 µl was plated on LA-cb plates and the rest shaken at 37 °C in 100 ml LB + 100 µl carbenicillin. After incubation overnight the library plasmid was extracted from this culture. Colonies appeared after incubation overnight (37 °C) and a small fraction was cultivated for individual minipreps and subsequent sequencing, verifying the diversity of the library.

Transformation to *Pichia pastoris*: *P. pastoris* X33 (Invitrogen) was made electrocompetent by adhering strictly to the manufacturer's protocol. Until employed, cells were stored in 40 μL aliquots at -80 °C without any treatment. Thawed X33 cells were mixed with library plasmid (1-3 μL) and electroporated (0.2 mm cuvettes, 1.5 kV, 1 pulse). Cells were incubated at 30 °C with YPDS (YPD with 1 M D-sorbitol, 1 mL) for 1-2 h followed by plating on YPD-agar plates containing zeocin (100 µg/mL), carbenicillin (100 µg/mL) and tributyrin (0.5 % v/v). After four days of incubation (30 °C), colonies expressing active lipase displayed a clear halo around them. Those colonies were picked and inoculated in a deep conical 96 well plates containing YPD media (1,1 mL). The plates were incubated in an orbital shaker (270 rpm, 30 °C, 8-10 days), where after they were harvested by centrifugation to obtain enzyme in the supernatant.

Organic solvent screening:

The enzyme supernatant was immobilized on Nunc Immobilizer Nickel-Chelate microtiter plates according to the manufacturers protocol. The plates were thereafter washed with isooctane (3 x 300 μL) to remove residual water. A screening solution (20 mM phenylethanol, 200 mM vinyl butyrate and 20 mM dodecane in isooctane) was premixed before addition to the plate using a multi-channel pipette. To each well was added 150 μL of the screening solution followed by incubation at 21 °C on a shaker. After 2.5 h a sample (13 μL) was removed from each well to a second microtiter plate containing 137 μL isooctane for GC-analysis. The plate was screened by chiral gas chromatography (GC-FID). The total time to screen through one 96-well plate was around 24 h, when using a method of 14.2 min. The GC-spectra from one plate is quickly overviewed by using an overlay view. Only chromatograms indicating hits (i.e. product) require integration. By further calculation, both

conversion and enantioselectivity can be determined. Variants showing an enantiomeric excess (ee_p) over 95 % were determined to be hits. In total, 17 hits were cultivated in 50 mL scale for further characterization (Table S1).

GC screen:

GC-analyses were performed using an IVADEX-1 chiral column. The screening was performed in 96 well microtiter plate format, where continuous integration and multi overlay of chromatograms simplified fast determination of possible hits. The GC program started at 90 °C (4 min), continued to 172 °C (10 °C/min) and then to 200 °C (100 °C/min, then hold for 2 min).

Sequencing hits:

Pelleted cells from master plates were used to inoculate YPD containing zeocin (100 µg/mL) and carbenicillin (100 µg/mL), and cultures were shaken at 30 °C over night. The plasmids were extracted using Yeast plasmid kit (Omega Bio-Tek), and subsequently transformed into *Escherichia coli* DH5α to obtain a higher plasmid yield. The bacterial cells were cultivated, plasmids were extracted and sequenced using the sequencing primerspBGP1_for (gtccctatttcaatcaattgaac) and pBGP1_rev (gtaagtgcccaacttgaactgag).

Exploring hit mutations:

TABLE S1. Conversion and enantiosselectivity shown by CalA variants found by library screening with product enantiomeric excess over 95% in kinetic resolution of the model reaction shown in Scheme 1.[a] Values are determined by chiral GC.

CalA variant[b]	Conversion (%)[c]	ee_p[d]	E[e] (R)	Mutation[f]
#4_25	4	99.0	203	Y93L/L367I/G387E
#8_17	13	98.6	143	Y93L/L367I
#8_84	22	98.1	135	Y93L/L367I
#8_20	16	98.3	133	Y93L/L367I
#8_69	30	98.2	127	Y93L/L367I
#8_81	22	98.2	124	Y93L/L367I
#4_30	8	98.2	124	Y93L/L367I
#8_67	29	98.0	119	Y93L/L367I
#8_50	24	97.8	119	Y93L/L367I
#8_94	26	97.7	111	Y93L/L367I
#8_92	32	97.7	108	Y93L/L367I
#8_93	23	97.7	108	Y93L/L367I
#8_91	29	97.8	105	Y93L/L367I
#7_59	51	95.4	93	Y93L/L367I

#7_29	6	97.4	81	Y93A/F233A/L367I
#1_29	18	96.7	60	Y93L/L367I
#3_29	28	95.8	50	Y93L/L367I

[a] Reaction condition: 200 mM vinyl butyrate, 20 mM 1-phenylethanol, 20 mM dodecane in isooctane at 21°C. The enzyme was immobilized on Nunc 96-well nickel-coated microtiter plate.
[b] The CalA variants are numbered according to the library number followed by the position in 96-well microtiter plate.
[c] The conversion is determined after 2.5 hours of reaction time by the use of dodecane as an internal standard and shown in percent.
[d] The enantioselectivity for the product (ee_p) is determined from GC analysis by ($A_{R(product)}$-$A_{S(product)}$)/($A_{R(product)}$+$A_{S(product)}$) and shown in percent.
[e] The *E*-value is determined according to the equation shown by Rakel *et al.*[10]
[f] Mutation determined by sequencing.

Table S2. Enantiospecificity of CalA wild-type (wt) and variant Y93L/L367I (LI) determined for kinetic resolution of various secondary alcohols by transacylation.[a]

Entry	Alcohol	Enzyme	Time (h)	Conversion (%)[b]	ee_s[c]	ee_p[c]	E[d]
1	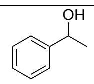	wt	2	24	12	51	3
		LI	0.7	34	7.8	98	100
2	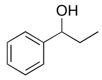	wt	4	15	16	60	5
		LI	4	6	7.3	91	20
3	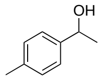	wt	2	5	5.6	94	30
		LI	3	13	21	>99	>300
4	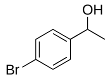	wt	2	45	71	94	70
		LI	2	41	69	>99	>300
5	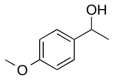	wt	5	3	18	93	30
		LI	5	2	10	91	20
6	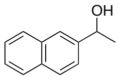	wt	3	33	19	96	70
		LI	3	15	7.7	98	100
7	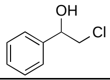	wt	1	8	5.9	60	4
		LI	1	7	4.7	98	100

[a] Reaction conditions: 200 mM vinyl butyrate, 20 mM 1-phenylethanol, 20 mM dodecane in isooctane at 21°C. The enzyme was immobilized on Nunc 96-well nickel-coated microtiter plate.
[b] The conversion is determined after 2.5 hours of reaction time by the use of dodecane as an internal standard and shown in percent.
[c] The enantioselectivity for the product (ee_p) is determined from GC analysis by ($A_{R(product)}$-$A_{S(product)}$)/($A_{R(product)}$+$A_{S(product)}$) and shown in percent.
[d] The *E*-value is determined according to the equation shown by Rakel *et al.*[10]

Sequencing hits:

Pelleted cells from master plates were used to inoculate YPD containing zeocin (100 µg/mL) and carbenicillin (100 µg/mL), and cultures were shaken at 30 °C over night. The plasmids were extracted using Yeast plasmid kit (Omega Bio-Tek), and subsequently transformed into *Escherichia coli* DH5α to obtain a higher plasmid yield. The bacterial cells were cultivated, plasmids were extracted and sequenced using the primers pBGP1_for (gtccctatttcaatcaattgaac) and pBGP1_rev (gtaagtgcccaacttgaactgag)

Determination of kinetic parameters, K_M and K_I:

Kinetic constants for wild-type CalA and Y93L/L367I were determined for 1-phenylethanol under pseudo-one substrate conditions using the model reaction screening conditions. Enzyme supernatant was immobilized on Nunc Immobilizer Nickel-Chelate microtiter plates according to the manufacturers protocol. The plates were thereafter washed with isooctane (3 x 300 μL) to remove residual water.

Solutions (3-100 mM 1-phenylethanol, 200 mM vinyl butyrate and 20 mM dodecane in isooctane) were premixed before addition to plate. To each well was added 150 μL of this solution followed by incubation at 21 °C on shaker. Samples were taken regularly (13 μL) to a second microtiter plate containing isooctane (137 μL) for GC-analysis. The kinetic constants were obtained by non-linear regression of the initial rates using the Michaelis-Menten equation for competitive substrate inhibition.

Exploring hit mutations by molecular modeling:

The effects of the mutation points on enantioselectivity were further explored by molecular modeling using a tetrahedral intermediate model of the model substrate in (*R*)- and (*S*)-configuration (Figure S1). The structure was prepared using the YASARA software[4]. Mutant Y93L/L367I and the two single-point mutants (Y93L and L367I) were created by swapping the specific amino acid residues. The structures were step-wise energy minimized, by firstly keeping all but the mutation point and the intermediate fixed and then free everything.

Both the (*R*)- or (*S*)-configurations of the tetrahedral intermediate in the different variant structures were compared by alignments to the corresponding ones in the wild-type enzyme using MUSTANG in the YASARA software. [4,11] The alignments showed that the configurations of the tetrahedral (*S*)- and (*R*)-intermediate phenyl groups were affected to different extent by the mutations. The mutations induce movements in the configuration of the tetrahedral intermediate phenyl groups due to steric hindrance. To evaluate to which extent each carbon in the phenyl groups have to move, the distance to its original position in the wild-type structure was measured. Table S3 shows the distance that each carbon in the phenyl group has to move, compared to its position in the wild-type structure, to find the proper orientation in the mutant structures. Each carbon in the tetrahedral intermediate phenyl group is numbered (C_{no}) as shown in Figure S1.

Mutation Y93L mainly affects the phenyl group of the tetrahedral intermediate in the (*S*)-configu-ration, while mutation L367I mainly affects the corresponding phenyl group in the (*R*)-configuration. In the double mutant, the phenyl group in the tetrahedral intermediate in the (*S*)-configuration has to move further compared to the corresponding one in the (*R*)-configuration. However, the important hydrogen bond network between the intermediate model structures and the oxyanion hole and His366 was not affected by the mutations.

However, the hydrogen bond length between the intermediate oxygen (OE) atom (originating from 1-phenylethanol) and His366 (HE2) proton were altered by the mutations. The same was true for the hydrogen bond angle between His366 nitrogen and hydrogen (NE2 and HE2) atoms and the transition state intermediate oxygen (OE) atom. The length of this hydrogen bond for the tetrahedral intermediate in the (*R*)-configuration is clearly reduced, while the length of the corresponding hydrogen bond for the tetrahedral intermediate in the (*S*)-configuration is only affected to a minor

extent (Table S4). The same hydrogen bond angles for the different mutations are increased for the tetrahedral intermediate in (*R*)-configuration, while same angles in the intermediate in (*S*)-configuration are decreased. According to the molecular modeling results, mutation Y93L/L367I affects the tetrahedral intermediate in (*R*)-configuration in a positive manner to a higher extent compared to the intermediate in (*S*)-configuration. These results confirm and follow the trend of the experimentally determined result for the three enzyme variants (Table 3 entry 1 and Table 4).

Figure S1. Tetrahedral intermediate model including the numbering of the phenyl group carbons (C_{no}).

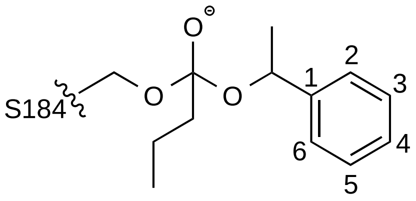

Table S3. Compiled distances (Å) that each carbon in the tetrahedral intermediate phenyl group in (*S*) and (*R*)-configuration has to move to find a proper orientation in the mutant structures compared to its position in the wild-type.

C_{no} [a]	Y93L$_R$ (Å)	Y93L$_S$ (Å)	L367I$_R$ (Å)	L367I$_S$ (Å)	Y93L/L367I$_R$ (Å)	Y93L/L367I$_S$ (Å)
1	0.350	0.669	0.923	0.075	0.767	0.687
2	0.271	1.690	1.399	0.078	1.263	1.686
3	0.206	2.079	1.756	0.090	1.508	2.067
4	0.207	1.530	1.630	0.106	1.268	1.437
5	0.247	0.927	1.231	0.113	0.843	0.705
6	0.332	0.671	0.922	0.093	0.604	0.582

[a] The carbon numbers (C_{no}) of each carbon in the tetrahedral intermediate phenyl group is shown in Figure S1.

Table S4. The hydrogen bond distances (Å) between His366 (HE2 atom) and the tetrahedral intermediate (TI) oxygen (OE atom) (originating from 1-phenylethanol) and the corresponding hydrogen bond angles (°) between NE2, HE2 and OE, in mutants and wild-type are shown.

Enzyme variant	Hydrogen bond distance (Å)		Hydrogen bond angle (°)	
	(R)-TI	*(S)-TI*	*(R)-TI*	*(S)-TI*
Wild-type	1.888	1.894	146.4	146.2
Y93L	1.835	1.878	150.4	139.8
L367I	1.807	1.888	149.3	146.4
Y93L/I367L	1.836	1.882	151.5	144.9

References:

[1] D. J. Ericsson, A. Kasrayan, P. Johansson, T. Bergfors, A. G. Sandstrom, J. E. Bäckvall,S. L. Mowbray, *J. Mol. Biol.* **2008**, *376*, 109.

[2] A. G. Sandström, K. Engström, J. Nyhlen, A. Kasrayan, J. E. Bäckvall, *Protein Eng., Des. Sel.* **2009**, *22*, 413.

[3] K. Engström, J. Nyhlén, A. G. Sandström, J. E. Bäckvall, *J. Am. Chem. Soc.* **2010**, *132*, 7038.

[4] E. Krieger, G. Koraimann, G. Vriend, *Proteins: Struct., Funct., Bioinf.* **2002**, *47*, 393.

[5] J. Wang, P. Cieplak, P. A. Kollman, *J. Comp. Chem.* **2000**, 21, 1049.

[6] U. Essmann, L. Perera, M. L. Berkowitz, T. Darden, H. Lee, L. G. Pedersen, *J. Chem. Phys*. **1995**, 103, 8577.

[7] C. C. Lee, T. G. Williams, D. W. S. Wong, G. H. Robertson, *Plasmid* **2005**, 54, 80-85.

[8] A. G. Sandström, Y. Wikmark, K. Engström, J. Nyhlen, J. E. Bäckvall, *Proc. Natl. Acad. Sci. USA* **2012**, 109, 78.

[9] C. T. Chung, S. L. Niemela, R. H. Miller, *Proc. Natl. Acad. Sci. USA,* **1989**, 86, 2172.

[10] J. L. L. Rakels, A. Straathof, J. J. Heijnen, *Enzyme Microb. Tech*. **1993**, 15, 1051.

[11] A. S. Konagurthu, J. C. Whisstock, P. J. Stuckey, A. M. Lesk, *Proteins: Struct., Funct., Bioinf.* **2006**, 64, 559.
